# Editorial: *In vitro* digestion in the study of food

**DOI:** 10.3389/fnut.2026.1785006

**Published:** 2026-02-02

**Authors:** Giacomo Di Matteo, Alessandra Cimbalo, Massimo Frangiamone

**Affiliations:** 1 Food Chemistry Laboratory, Department of Chemistry and Technology of Drugs, Sapienza University of Rome, Rome, Italy; 2 Laboratory of Food Chemistry and Toxicology, Faculty of Pharmacy, Universitat de València, Valencia, Spain; 3 Department of Biomedical Sciences, University of Lausanne, Lausanne, Switzerland

**Keywords:** bioaccessibility, duodenum, dynamic digestion, *in vitro* digestion, static digestion

*In vitro* digestion models are widely used to study how foods and pharmaceuticals behave along the gastrointestinal tract. While human nutrition studies remain the gold standard, simulated digestion is faster, cheaper, less labor-intensive, and free of ethical constraints, enabling parallel screening and highly controlled, reproducible mechanistic work with easy sampling at specific sites. The international INFOGEST network has played a significant role in this area, particularly in standardizing and validating static *in vitro* digestion protocols and correlating these findings with *in vivo* data (1–3). These standardized *in vitro* digestion protocols are essential for the assessment of the gastrointestinal fate of foods under controlled conditions and focus on matrix-driven release, along with the stability and bioaccessibility of nutrients and bioactives. Their applications now span macronutrient digestibility, the post-digestive behavior of phytochemicals (e.g., polyphenols and carotenoids), and the production of bioaccessible fractions. By reducing methodological variability, harmonized protocols (INFOGEST-like) strengthen cross-study comparability while remaining adaptable to different food matrices and research aims (4, 5). Moreover, digested foods can be used in downstream mechanistic assays, such as epithelial transport models (6).

Yikmiş et al. applied INFOGEST *in vitro* gastrointestinal digestion (followed by dialysis) to determine if processing improved bioactive delivery in dill (*Anethum graveolens*) juice beyond simple retention. Comparing control, pasteurized, and thermosonicated juices, the authors quantified the post-digestion bioaccessibility of phenolics, β-carotene, chlorophyll, and FRAP activity, and related these outcomes to GC–MS aroma profiles. Thermosonication yielded the highest post-digestion levels of key bioactives and was associated with aroma–function correlations, while also preserving volatile stability better than conventional heating. Overall, the authors concluded that thermosonication enhances the antioxidant quality and post-digestion bioaccessibility of dill juice bioactives. Bietto et al. illustrated a complementary application of *in vitro* digestion in early-life nutrition research by using it to generate infant formula digesta. The digesta was then used to study the crossing of an infant-like intestinal barrier in a controlled cell model. The authors developed an *in vitro*, temporary, infant-like gut barrier model, treating Caco-2/HT29-MTX monolayers with sodium glycodeoxycholate (GDC). The infant milk was digested using the INFOGEST protocol, the enzyme action was stopped by adding inhibitor enzymes, and the digesta was added to the apical compartment of the Caco-2/HT29-MTX monolayers, both with and without GDC treatment, for 2 h, recording TEER values. GDC reduced TEER and increased paracellular permeability in Caco-2/HT29-MTX monolayers without causing inflammation or cytotoxicity. Applying *in vitro*–digested infant formula to 0.8 mM GDC-treated Caco-2/HT29-MTX monolayers increased basolateral amino acid concentrations compared with untreated control monolayers. The authors concluded that GDC can modulate gut barrier properties, providing a valuable *in vitro* model to study early-life gut physiology and nutrient absorption. Di Matteo et al. used *in vitro* digestion to estimate the bioaccessibility of botanical phytochemicals and also to create physiologically relevant digesta for intestinal barrier and toxicity-mitigation assays. First, the authors chemically characterized wild *Gentiana lutea* flowers using HPLC-PDA and NMR metabolomics, identifying abundant bioactive compounds (iridoids, secoiridoids, and xanthones) alongside primary metabolites (amino acids, organic acids, and sugars), and then applied the INFOGEST protocol. The digesta were frozen at −80 °C in order to stop the digestion process and subsequently analyzed for (i) the bioaccessibility of key secondary metabolites and (ii) the transepithelial transport across differentiated Caco-2 monolayers. In Caco-2 cells, the digested *Gentiana lutea* flower fraction reduced AFB1-, OTA-, and BEA-induced toxicity by lowering pro-apoptotic markers (BAX and CASP3), supporting tight-junction genes (CL-2 and ZO-1), and enhancing antioxidant responses (SRXN1). Overall, *Gentiana lutea* flowers emerged as a potential source of phytocompounds for mycotoxin-related barrier protection.

Pessotti et al. then addressed the next downstream stage of the gastrointestinal process, focusing on how food-derived substrates that reach the colon are metabolized by the gut microbiota. This shapes both fermentation products and the composition of the microbial community. The authors investigated the colonic microbiota's response to two synbiotic yogurts, using an *in vitro* dynamic multivessel colon simulation (xGIbiomics^®^ technology) that mimics human gut conditions. This technology consists of five sequentially connected reactors that mimic the stomach, small intestine, ascending colon, transverse colon, and descending colon. For this procedure, they referred to the INFOGEST protocol for pre-conditioning the yogurt dose with a simulated salivary solution and a pepsin-containing gastric step before introducing it into the xGIbiomics^®^ system.

Another aspect of digestion involves the utilization of enzymatic digestion to generate potentially bioaccessible fractions, which can then be evaluated for biological relevance. Yang et al. used enzymatic digestion to produce quinoa protein hydrolysates and assess how protease choice shaped antioxidant potential. The authors compared four enzymes, including the digestive proteases pepsin and trypsin alongside industrial proteases, linking hydrolysis features (degree of hydrolysis and low-MW peptides) to antioxidant assays and HepG2 cytoprotection. This positions enzymatic digestion as an upstream step for generating bioactive pools relevant to health food product applications.

In addition to experimental studies modeling gastrointestinal fate through simulated digestion and colonic fermentation platforms, Semchyshyn reviewed dietary glycation products as a paradigmatic case in which limited small intestinal absorption and extensive microbial processing in the colon jointly shape physiological exposure and downstream health effects. The author emphasized that a substantial fraction of ingested glycotoxins may escape small-intestinal uptake, thereby interacting with the gut microbiota and potentially influencing both microbial ecology and systemic outcomes. While high exposure has been associated with inflammation and oxidative/carbonyl stress, the review also discussed the possibility of adaptive effects at lower levels and highlighted major knowledge gaps in defining “physiological” exposure ranges and host–microbe contributions to glycotoxin handling.

In summary, the studies gathered in this Research Topic provide a diverse and timely set of examples showing how simulated digestion, when coupled with food processing, intestinal cell models, and microbiota-focused platforms, can translate food composition into more mechanistic and physiologically meaningful insights. Collectively, these contributions reinforce the idea that *in vitro* approaches can move beyond descriptive compositional data to examine bioaccessibility, epithelial responses, and downstream microbial metabolism. After reading this Research Topic, readers should find the value of standardized yet adaptable digestion workflows clearer and be further convinced that integrating digestion with downstream intestinal and microbiome models is a fundamental step toward more robust, comparable, and actionable nutrition research.
